# A nutrient pattern characterized by vitamin A, C, B6, potassium, and fructose is associated with reduced risk of insulin‐related disorders: A prospective study among participants of Tehran lipid and glucose study

**DOI:** 10.1186/s13098-021-00629-4

**Published:** 2021-01-26

**Authors:** Farshad Teymoori, Ebrahim Mokhtari, Pantea Salehi, Firoozeh Hosseini-Esfahani, Parvin Mirmiran, Fereidoun Azizi

**Affiliations:** 1 Nutrition and Endocrine Research Center, Research Institute for Endocrine Sciences, Shahid Beheshti University of Medical Sciences, P.O. Box: 1985717413, Tehran, Iran; 2 Department of Nutrition, School of Public Health, Iran University of Medical Sciences, Tehran, Iran; 3 Department of Clinical Nutrition and Dietetics, Faculty of Nutrition and Food Technology, Shahid Beheshti University of Medical Sciences, Tehran, Iran; 4 Endocrine Research Center, Research Institute for Endocrine Sciences, Shahid Beheshti University of Medical Sciences, Tehran, Iran

**Keywords:** Nutrient pattern, Insulin resistance, Insulin insensitivity, Insulinemia, Principal Component analysis

## Abstract

**Background:**

Insulin-related disorders, including insulin resistance, insulin insensitivity, and insulinemia, is considered early predictors of major chronic disease risk. Using a set of correlated nutrient as nutrient patterns to explore the diet-disease relationship has drawn more attention recently. We aimed to investigate the association of nutrient patterns and insulin markers’ changes prospectively among adults who participated in the Tehran Lipid and Glucose Study (TLGS).

**Methods:**

For the present study, 995 men and women aged 30–75 years, with complete information on insulin and dietary intake in survey III TLGS, were selected and followed three years until survey IV. Dietary intakes at baseline were assessed using a valid and reliable food frequency questionnaire (FFQ). Nutrient patterns were derived using principal component analysis (PCA). We extracted five dominant patterns based on the scree plot and categorized them into quartiles. Linear regression analysis was conducted to investigate the association between Nutrient patterns and three-year insulin markers changes, including fasting insulin, HOMA-IR, and HOMA-S.

**Results:**

The mean (SD) age and BMI of participants (43.1 % male) were 46.2(10.9) year and 28.0(4.7) kg/m^2^, respectively. The median (IQR, 25, 75) of 3 years changes of insulin, HOMA-IR and HOMA-S were 0.35 (− 1.71, 2.67) mU/mL, 0.25 (− 0.28, 0.84) and − 6.60 (− 22.8, 7.76), respectively. In the fully adjusted model for potential confounders, per each quartile increment of the fifth nutrient pattern, the β coefficients (95 % CI) of changes in insulin, HOMA-IR, and HOMA-S were − 0.36 (− 0.62, − 0.10); P value = 0.007, -0.10 (-0.19, -0.01); P value = 0.022, and 1.92 (0.18, 3.66); P value = 0.030, respectively. There were no significant association between other nutrient patterns and insulin related indices.

**Conclusions:**

Present study showed that high adherence to a nutrient pattern rich in vitamin A, vitamin C, pyridoxine, potassium, and fructose is inversely associated with 3-years changes in insulin, HOMA-IR, and directly associated with HOMA-S.

## Background

Insulin-related disorders, including insulin resistance (IR), insulin insensitivity, and hyperinsulinemia considered early predictors of major chronic diseases such as diabetes mellitus (T2DM), metabolic syndrome (MetS), and cardiovascular risk [1–3]. An unhealthy diet has been known as one of the most important modifiable risk factors of these disorders, along with obesity, smoking, sedentary lifestyle, etc. [4, 5].

Using dietary patterns in nutritional researches has drawn more attention over the past decades. This method provides a better assessment of the relationship between nutrition status and risk of diseases rather than individual foods or nutrients [6]. However, dietary patterns face some limitations. For example, they are unable to explain the mechanisms by which they affect disease development. Their nutrients apply to the food effects, and despite foods, nutrients are identical all over the world and not affected by cooking or preservation methods [7]. So, nutrients can be considered as promising targets to use in nutrition investigations. Accordingly, the association between several nutrients and insulin-related disorders have been explored in previous studies. However, it is demonstrated that a combination of different nutrients that form a nutrient pattern is more predictive about the diet-disease association than an individual nutrient because that includes a complex of interrelationships between several nutrients represents their collective effects [8]. In this approach, firstly, the intake of nutrients is determined via food composition databases, and then the set of nutrients with higher correlation forms nutrient patterns using statistic methods [9].

Several studies investigated the association between nutrient patterns and chronic diseases. Vajdi et al. recently conducted a study exploring the relationship of nutrient patterns and MetS and found that greater adherence to the pattern composed of fiber, carbohydrate, vitamins D, B6, B3, C, B1, E, magnesium, potassium, linoleic acid, and docosahexaenoic acid (DHA) is negatively associated with the risk of MetS. In contrast, animal- and mixed-sourced nutrient patterns directly associate with MetS risk [10]. Another study extracted three nutrient patterns related to central obesity in adults; a pattern characterized by thiamine, niacin, betaine, folate, iron, selenium, and starch was associated with decreased risk of central obesity while another pattern including glucose, fructose, sucrose, fiber, C and K vitamins, and copper increased the risk [9]. Besides, the association of two nutrient patterns characterized by animal-derived nutrients and starch and folate with higher body mass index was observed in Pisa et al. [11]. Also, various nutrient patterns related to some chronic diseases and biomarkers like hemoglobin A1c (HbA1c) and fasting blood sugar (FBS) were obtained in other surveys [8, 12, 13]. We previously extracted five nutrient patterns related to cardiometabolic factors and found a pattern rich in fructose, vitamins A, C, pyridoxine, and potassium associated with lower triglyceride level [14].

The insulin-related disorders, especially IR, are the key underlying factor for a wide range of chronic diseases; however, there is no study concerning their relation with diet at the level of nutrient patterns. Regarding the paucity of evidence, the present study aimed to investigate the association of nutrient patterns and changes in insulin homeostasis related markers, including fasting insulin concentration, HOMA-IR, and HOMA-S, prospectively among adults participated in the Tehran Lipid and Glucose Study (TLGS).

## Methods

### Study participants

**Fig. 1:**
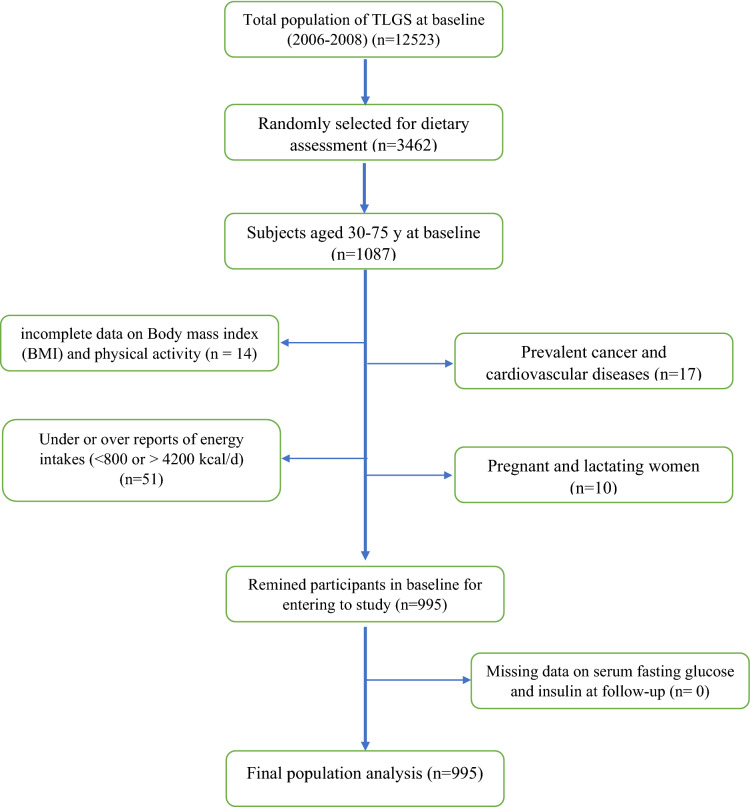
The diagram of the study participants and follow-up

The present study conducted in the framework of TLGS, which is a prospective study aimed to determine the prevalence and identifying the risk factors of chronic diseases, began in 1999–2001 on 15,005 participants, aged ≥ 3 years, on residents of district No. 13 of Tehran, the capital of Iran. The participants have followed up every three years to update their demographics and lifestyle, biochemical, clinical, and dietary information. In the third survey of the TLGS (2006–2008), of 12 523 participants, 3462 were randomly selected for dietary assessment. For the present study, 1087 men and women aged 30–75 were selected with complete information on insulin and dietary intake. Subjects with a history of myocardial infarction or stroke and cancer (n = 17), those who reported daily energy intakes outside the range of 800–4200 kcal/ day (n = 51), those on incomplete data on Body mass index (BMI) and physical activity (n = 14), and pregnant and lactating women (n = 10) were excluded. Finally, 995 participants were followed until Survey IV (2009–2011), and data analysis was conducted on this population (Fig. 1).

**Fig. 2:**
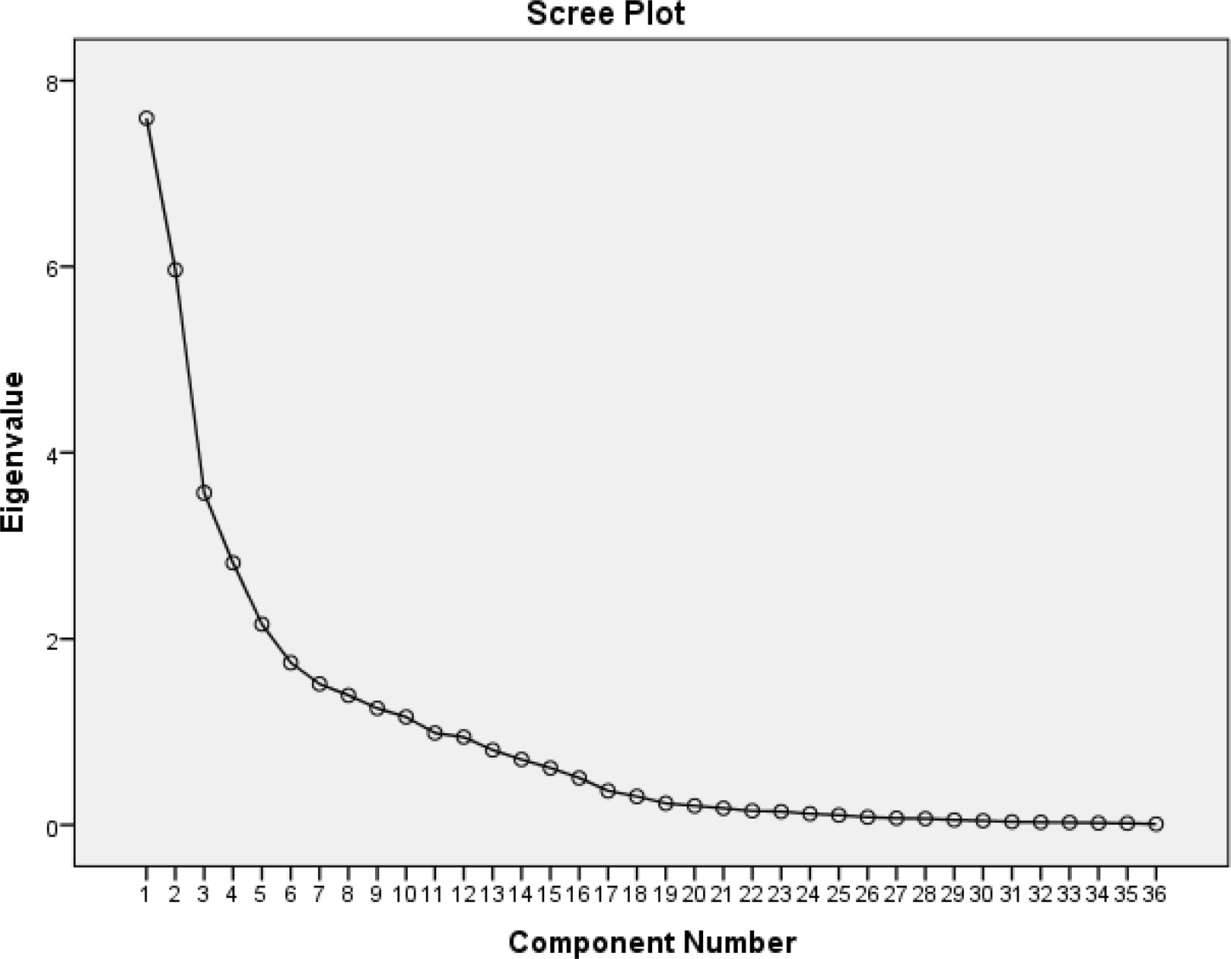
Scree plot for extraction of dietary nutrient patterns by principal component analysis. The 36 dietary nutrients was used as input variables and nutrient patterns based on eigenvalues > 2 were identified as main nutrient patterns

The proposal of this study was approved by the Ethics Committee of the Research Institute for Endocrine Science, Shahid Beheshti University of Medical Sciences, Tehran, Iran. Written informed consent was obtained from all participants.

### Dietary intake assessment

Dietary intakes were assessed using a valid and reliable semi-quantitative food frequency questionnaire (FFQ). The reliability and validity of the FFQ have been previously reported [15]. During a face-to-face interview, the consumption frequency for each food item during the previous year on a daily, weekly, or monthly basis of participants was collected by trained and experienced dieticians. Portion sizes of consumed foods reported in household measures were then converted to grams. Energy and nutrient contents were computed using the United States Department of Agriculture (USDA) food composition table (FCT). For local food items that were not available in USDA FCT, the Iranian FCT was used. During the third phase (2009–2011), dietary intake assessment of TLGS were considered dietary intake exposure at baseline.

### Physical activity assessment

The modifiable activity questionnaire, used for assessing physical activity levels in participants, modified and validated among Iranians, previously [16]; individuals were asked to report and identify the frequency and time spent during the past 12 months on activities of light, moderate, hard, and very hard intensity, according to a list of common activities of daily life; physical activity levels are expressed as metabolic equivalent hours per week (Met.h.wk).

### Clinical and biological measurements

Information on age, sex, medical history, medication use, and participants’ smoking habits were collected by trained interviewers using pretested questionnaires. Weight and height were measured using standard protocols. BMI was computed as weight in kilograms, divided by height in meters squared.

All subjects’ blood samples were collected after 12 to 14 hours of overnight fast in a sitting position between 7:00 and 9.00 AM., immediately centrifuged within 30–45 min of collection. All blood samples were analyzed at the TLGS research laboratory on the day of blood collection using Selectra 2 auto-analyzer (Vital Scientific, Spankeren, Netherlands). FBS was measured using an enzymatic colorimetric method with glucose oxidase. Inter- and intra-assay coefficient variations for FBS were both 2.2 % for FBS. Fasting Insulin was measured via electrochemiluminescence immunoassay (ECLIA), using Roche Diagnostics kits and Roche/Hitachi Cobas e-411 analyzer (Gmbh, manhim, Germany). Inter- and intra-assay coefficient variations for insulin were 1.2 and 3.5, respectively.

Homeostatic model assessment for insulin resistance (HOMA-IR) and Homeostatic model assessment for insulin sensitivity (HOMA-S) was calculated using the following formula:

HOMA-IR = FBS (mmol/L) × Insulin (µU/mL) / 22.5, HOMA-S = (1 / HOMA-IR) × 100.

Diabetes is a metabolic disease characterized by hyperglycemia resulting from insufficient insulin secretion in pancreatic β-cells, insulin action in peripheral tissues, or both.

Clinically, a diabetic patient is defined as an individual with at least one of the following criteria:

FBS ≥ 126 mg/dL (7.0 mmol/L), 2-hBS ≥ 200 mg/dL (11.1 mmol/L), consumption of blood glucose lowering medications, or all of them [17].

### Statistical analysis

Data analyses were conducted using Statistical Package for Social Sciences (version 20.0; SPSS Inc, Chicago IL). The normality of variables assessed using histogram charts and Kolmogorov–Smirnov test. Kruskal-Wallis test was used for non-normal variables.

The 36 nutrients were selected for factor analysis, including Starch, Sucrose, Lactose, Fructose, Glucose, Animal protein, Plant protein, Fibre, Saturated fatty acids (SFA), monounsaturated fatty acids (MUFA), Polyunsaturated fatty acids (PUFA), cholesterol, Vitamin A, Vitamin D, Vitamin E, Vitamin K, Thiamine, Riboflavin, Niacin, Pantothenic acid, Pyridoxine, Folate, Vitamin B12, Vitamin C, Calcium, Phosphor, Iron, Zinc, Copper, Magnesium, Manganese, Chromium, Selenium, Sodium, Potassium, and Caffeine. Nutrient patterns were derived using principal component analysis (PCA) with varimax rotation and based on the correlation matrix. Statistical correlation between variables and adequacy of sample size was tested, using the Bartlett test of sphericity (P < 0.001) and the Kaiser-Mayer-Olkin test (0.81). Factor scores for all participants for each of the extracted factors were calculated by summing the frequency of consumption, multiplied by factor loadings across all 36 nutrient items. We identified five dominant factors based on the scree plot (eigenvalue > 1) and categorized them into quartiles cut off points.

Baseline characteristics of subjects were expressed as mean ± SD or median (25–75 interquartile range) for continuous variables and percentage for categorical variables across quartiles of nutrient patterns. The three-year changes of Insulin, HOMA-IR, and HOMA-S were ascertained by subtracting those values of survey four from survey three. Multiple linear regression analysis was conducted with Insulin, HOMA-IR, and HOMA-S changes as the dependent variable and quartiles of nutrient patterns as independent continuous variables.

The analysis was adjusted for potential confounders, including age, sex, BMI, physical activity, smoking, daily energy intake, education levels, marital and employment status, diabetes status, and family history of diabetes. Furthermore, for each of the dependent variables, its value in the baseline phase was adjusted. Beta coefficient (unstandardized) and their respective confidence intervals 95 % (95 % CI) were reported, and P-values < 0.05 were considered as statistically significant.

## Results

The age and BMI mean (SD) of participants (43.1 % male) were 46.2(10.9) years and 28.0(4.7) kg/m^2^, respectively. The median (IQR, 25, 75) of 3 years changes of insulin, HOMA-IR and HOMA-S were 0.35 (− 1.71, 2.67) mU/mL, 0.25 (-0.28, 0.84) and − 6.60 (− 22.8, 7.76), respectively.

The factor loading matrix of 36 nutrient intakes and variances of each of five nutrient patterns were shown in Table 1. Using the principal component analysis (PCA) method, five dominant patterns (Fig. 2) were ascertained, which explained 62.2 % of total variations of 36 nutrient intakes. The first pattern covered 22 % of the total variance; plant proteins, thiamine, niacin, phosphorus, zinc, copper, magnesium, manganese, and selenium had the highest factor loading in this pattern. The second pattern is characterized by animal protein, lactose, vitamin D, riboflavin, pantothenic acid, vitamin B12, calcium, phosphorus, and zinc. Vitamin K, fiber, calcium, iron, manganese, and potassium were highly loaded in the third pattern. The fourth pattern had a positive correlation with starch, thiamine, folate, and a high negative correlation with mono and polyunsaturated fatty acids and vitamin E. The fifth pattern has the highest factor loading for fructose, vitamins A and C, pyridoxine, and potassium.

Table 2 indicates the general characteristics of participants based on quartiles of nutrient patterns. Across quartiles of the first pattern, the mean age, male percentage, insulin, FBS, HOMA-IR, diabetic participants, and those who were consumed glucose-lowering drugs increased (P < 0.05). The male percentage decreased for the second pattern, and diabetic participants increased (P < 0.05). With increasing the third pattern score, the mean change of insulin and HOMA-IR increased, and HOMA-S decreased (P < 0.05). The percentage of men and smokers across quartiles of the fourth pattern increased; whereas, the percentage of men, smokers, and the mean change of HOMA-S across the fifth pattern decreased, and the mean age increased (P < 0.05).

Food group and macronutrient intakes across quartiles of nutrient patterns are presented in Table 3 With increasing quartiles of the first pattern, carbohydrate, protein, fiber, grain, vegetable, white meat, and legume intakes increased (P < 0.001); whereas, energy intake, fat, fruit, dairy, and simple sugar consumptions decreased (P < 0.001). Across quartiles of the second pattern, energy intake, protein, fat, dairy, and legume consumption increased (P < 0.05); however, the intake of carbohydrates, fiber, grain, vegetables, and simple sugar decreased (P < 0.05).

Dietary protein, fiber, vegetables, dairy increased across quartiles of the third pattern (P < 0.05); whereas, energy intake, carbohydrate, fruit, grain, and simple sugar intakes decreased (P < 0.05). By increasing the score of the fourth pattern, intake of carbohydrate, protein, fiber, and grain elevated (P < 0.05); however, fat, vegetable, and legume reduced (P < 0.05). Dietary intake of carbohydrate, protein, fiber, fruit, vegetable, and legume increased across quartiles of the fifth pattern (P < 0.05); whereas, intake of energy, fat, grain, dairy, red meat, and simple sugar decreased (P < 0.001).

The association between nutrient patterns and 3-year changes in plasma insulin, HOMA-IR, and HOMA-S among Iranian adults of the TLGS study is demonstrated in Table 4. In the age and sex-adjusted model, per each quartile increment of the first nutrient pattern, the beta (β) coefficient (95 % CI) of HOMA-IR change was 0.09 (0.01, 0.02); P-value = 0.03. Also, in model 2, after further adjustments, the association remained significant. However, in the fully adjusted model after adjusting for diabetes status at baseline and family history of diabetes, the beta (95 % CI) became 0.08 (-0.01–0.19); P-value = 0.094, and the association lost its significance. The first pattern showed no significant association with changes in insulin and HOMA-S.

**Table 1:** Factor loading matrix and explained variances for major nutrient patterns identified by factor analysis^a,b^

	Nutrient patterns				
Nutrients	Pattern 1	Pattern 2	Pattern 3	Pattern 4	Pattern 5
Starch				*0.40*	
Sucrose					
Lactose		*0.90*			
Fructose					*0.55*
Glucose					
Animal protein		*0.52*			
Plant protein	*0.80*				
Fiber			*0.40*		
Saturated fatty acids					
Mono unsaturated fatty acids				*-0.84*	
Poly unsaturated fatty acids				*-0.83*	
cholesterol					
Vitamin A					*0.64*
Vitamin D		*0.73*			
Vitamin E				*-0.78*	
Vitamin K			*0.94*		
Thiamine	*0.55*			*0.53*	
Riboflavin		*0.75*			
Niacin	*0.52*	*-0.32*			
Pantothenic acid		*0.64*			0.38
Pyridoxine			0.32		*0.46*
Folate			0.36	*0.58*	
Vitamin B12		*0.41*			
Vitamin C					*0.86*
Calcium		*0.71*	*0.64*		
Phosphor	*0.42*	*0.72*			
Iron			*0.93*		
Zinc	*0.60*	*0.42*	0.32		
Copper	*0.66*				
Magnesium	*0.75*				
Manganese	*0.66*		*0.51*		
Chromium					
Selenium	*0.88*				
Sodium					
Potassium		0.38	*0.55*		*0.67*
Caffeine					
Explained variance (%)	22.0	17.2	9.5	6.8	6.4
Cumulative explained variance (%)	22.0	39.3	48.8	55.7	*62.2*

^a^Principle Component Analysis (PCA) performed on 36 nutrients adjusted for total energy intake

Nutrients with loadings > 0.40 and less than − 0.40 (in italics) are being characteristic for the five patterns; loadings less than 0.3 (in absolute value) are suppressed

^b^Kaiser’s Measure of Sampling Adequacy, KMO = 0.70, Bartlett’s test of sphericity = < 0.001

**Table 2:** Baseline characteristics of 995 participants 30-75 year old across quartiles of nutrient patterns^a^ (n = 995)

	Pattern 1		Pattern 2		Pattern 3		Pattern 4		Pattern 5	
	Q 1 (n = 244)	Q 4 (n = 259)	Q 1 (n = 244)	Q 4 (n = 259)	Q 1 (n = 242)	Q 4 (n = 256)	Q 1 (n = 246)	Q 4 (n = 252)	Q 1 (n = 229)	Q 4 (n = 273)
Age (years)	45.3 ± 10.7	47.9 ± 11.2^‡^	45.8 ± 10.1	46.7 ± 11.3	45.4 ± 11.0	46.4 ± 11.4	45.3 ± 9.9	46.9 ± 11.8	45.2 ± 10.5	47.9 ± 11.4^†^
Men (%)	37.1	51.5^‡^	50.5	37.8^‡^	49.8	40.5	33.2	59.5^†^	53.5	37.9^‡^
Body mass index (kg.m2)	27.9 ± 4.4	28.3 ± 5.1	27.9 ± 4.8	28.0 ± 4.6	27.8 ± 4.8	27.8 ± 4.8	27.9 ± 4.6	27.4 ± 4.8	27.4 ± 4.2	28.4 ± 4.7
Smoking (%)	16.1	12.3	17.0	11.1	14.0	12.9	11.7	17.9^‡^	18.0	9.0^†^
Physical activity (MET.h.wk)	27.3 (12.2–57.6)	27.7 (11.4–58.0)	27.8 (10.4–65.9)	27.7 (11.6–64.0)	27.8 (12.1–68.8)	27.8 (12.5–57.6)	27.3 (12.5–55.0)	27.8 (11.9–61.0)	27.8 (11.8–58.2)	26.5 (12.4–57.9)
Marital status (% of married)	62.9	56.7	59.9	63.0	64.3	59.5	65.2	58.8	59.0	56.2
Education levels (higher than diploma)	30.8	30.2	26.7	30.9	35.3	30.3	30.5	31.3	30.9	28.3
Employment status (employed, %)	70.5	64.6	71.3	71.4	73.6	70.5	72.7	67.9	68.2	63.8
Diabetes status (%)	4.5	14.9^†^	4.9	11.5^‡^	6.8	9.8	9.0	8.8	6.9	11.4
Glucose lowering drug consumption (%)	3.1	9.0^†^	3.6	7.3	4.7	6.8	5.5	5.0	4.1	6.9
Family history of diabetes (%)	16.1	16.8	15.0	18.7	20.0	17.4	18.0	14.1	14.3	16.9
Biochemical factors										
Insulin (μU/mL)	8.7 ± 5.2	9.6 ± 7.4^‡^	9.8 ± 6.5	9.2 ± 6.5	8.6 ± 4.7	9.9 ± 7.5^‡^	8.5 ± 4.6	9.5 ± 6.0	9.0 ± 6.3	9.5 ± 6.3
Fasting Blood Sugar (mg/dl)	89.5 ± 19	99.1 ± 34.3^†^	94.1 ± 26.9	96.1 ± 32.1	92.1 ± 22.8	94.8 ± 30.0	93.4 ± 26.8	95.5 ± 27.3	93.9 ± 29.2	96.1 ± 30.4
HOMA-S	71.1 ± 40.5	66.0 ± 46.3	65.9 ± 53.7	64.0 ± 36.1	70.5 ± 50.3	62.0 ± 36.3^‡^	72.9 ± 54.6	64.3 ± 40.8	72.7 ± 55.5	62.5 ± 36.5^‡^
HOMA-IR	2.0 ± 1.5	2.5 ± 3.3^‡^	2.4 ± 2.3	2.4 ± 3.1	2.04 ± 1.7	2.5 ± 3.2^‡^	2.0 ± 1.7	2.3 ± 2.0	2.3 ± 2.9	2.4 ± 2.7

^a^Data represented as mean ± SD or median (interquartile 25–75) and percent

P for trend: ^†^ < 0.001; ^‡^ < 0.05. P trend was calculated using general linear models for continuous variables or Chi square tests for categorical variables

**Table 3:** Dietary intakes in 995 participants 30–75 year old across quartiles of nutrient patterns^a^

	Pattern 1		Pattern 2		Pattern 3		Pattern 4		Pattern 5	
	Q 1 (n = 244)	Q 4 (n = 259)	Q 1 (n = 244)	Q 4 (n = 259)	Q 1 (n = 242)	Q 4 (n = 256)	Q 1 (n = 246)	Q 4 (n = 252)	Q 1 (n = 229)	Q 4 (n = 273)
Energy (kcal)	2562 ± 659	2312 ± 708^†^	2095 ± 696	2412 ± 668^†^	2460 ± 761	2344 ± 698^‡^	2363 ± 714	2360 ± 723	2566 ± 723	2308 ± 710^†^
Carbohydrates (% of energy)	55.5 ± 8.2	60.6 ± 6.5^†^	59.6 ± 8.3	56.3 ± 6.8^†^	59.6 ± 8.3	56.3 ± 6.8^‡^	52.3 ± 6.7	63.7 ± 6.1^†^	56.3 ± 8.1	60.1 ± 6.7^†^
Total protein (% of energy)	12.2 ± 2.0	15.0 ± 2.3^†^	12.5 ± 2.6	14.9 ± 2.0^†^	13.1 ± 2.2	14.4 ± 2.3^†^	12.8 ± 2.3	13.9 ± 2.3^†^	13.4 ± 2.3	13.9 ± 2.6^‡^
Total fat (% of energy)	34.6 ± 7.9	27.7 ± 5.5^†^	30.3 ± 8.2	31.5 ± 6.2^‡^	29.9 ± 7.5	30.6 ± 6.6	37.9 ± 6.5	24.6 ± 4.8^†^	31.8 ± 8.2	29.3 ± 6.2^†^
Fibre (g/1000 kcal)	15.1 ± 6.4	19.7 ± 7.1^†^	17.7 ± 8.2	16.1 ± 6.3^‡^	14.8 ± 5.0	21.5 ± 8.7^†^	15.1 ± 5.9	19.6 ± 10.1^†^	17.7 ± 10.1	19.0 ± 5.9^‡^
Grains (g/d)	412 ± 170	522 ± 255^†^	481 ± 262	417 ± 207^‡^	508 ± 282	445 ± 209^†^	386 ± 199	585 ± 288^†^	610 ± 289	376 ± 174^†^
Fruits (g/d)	445 ± 371	355 ± 270^†^	336 ± 272	373 ± 289	440 ± 339	381 ± 291^‡^	380 ± 189	580 ± 305	173 ± 106	636 ± 347^†^
Vegetables (g/d)	310 ± 178	345 ± 261^‡^	268 ± 181	351 ± 232^†^	288 ± 186	394 ± 204^†^	330 ± 233	291 ± 199^‡^	211 ± 114	445 ± 249^†^
Dairy (g/d)	582 ± 331	447 ± 256^†^	201 ± 124	769 ± 299^†^	476 ± 322	516 ± 295^‡^	433 ± 255	490 ± 327	529 ± 344	480 ± 284^‡^
Red and processed meat (g/d)	28.5 (16.6–43.1)	21.7 (12.4–40.1)	25.3 (13.9–40.4)	23.5 (14.1–37.0)	24.0 (13.1–39.0)	24.0 (14.8–37.4)	26.0 (15.5–42.6)	21.0 (12.2–36.7)	28.4 (13.8–46.3)	23.1 (13.7–36.8)^‡^
White meats (g/d)	29.6 (18.0–44.7)	33.0 (18.5–51.2)^‡^	28.4 (16.0–47.6)	29.6 (19.0–47.0)	30.7 (19.2–50.4)	32.2 (18.0–50.8)	29.0 (16.7–43.1)	28.5 (18.6–46.3)	27.7 (15.7–44.5)	31.8 (20.0–50.0)
Legume and nuts (g/d)	16.4 (10.1–26.32)	20.0 (10.4–38.0)^†^	13.9 (7.5–26.4)	17.8 (11.1–32.3)^‡^	17.1 (9.7–31.4)	19.9 (10.9–36.0)	18.3 (11.6–32.2)	14.0 (8.7–26.1)^‡^	14.7 (7.9–26.9)	19.8 (11.4–33.9)^‡^
Sweets and simple sugars	87.5 (56.1–148.5)	46.6 (22.9–75.3)^†^	63.3 (39.8–106.3)	53.9 (30.3–85.8)^‡^	67.4 (38.6–122.1)	50.2 (29.1–80.9)^†^	61.6 (37.2–109.0)	60.8 (31.1–95.6)	66.9 (40.2–108.1)	49.6 (23.8–87.0)^†^

^a^Data represented as mean ± SD or median (interquartile 25–75) and percent

P for trend: ^†^ < 0.001; ^‡^ < 0.05. P trend was calculated using general linear models

**Table 4:** Beta coefficient and 95% CI of changes in insulin indices per each quartile increase of nutrient patterns

	Insulin changes		HOMA_IR changes		HOMA-S changes	
	Β^a^ (95% CI)	*P* value	Β(95% CI)	P-value	Β(95% CI)	P-value
Pattern 1						
Model 1^b^	0.21 (−0.04–0.47)	0.108	0.09 (0.01–0.18)	0.028	−1.58 (−3.34–0.17)	0.078
Model 2^c^	0.23 (−0.03–0.49)	0.081	0.09 (0.01 –0.18)	0.030	−1.47 (−3.22–0.26)	0.097
Model 3^d^	0.23 (−0.07 –0.53)	0.137	0.08 (−0.01–0.19)	0.094	−1.46 (−3.19–0.26)	0.097
Pattern 2						
Model 1^b^	−0.00 (−0.26–0.25)	0.962	−0.00 (−0.09–0.08)	0.947	−0.50 (−2.23–1.23)	0.571
Model 2^c^	−0.04 (−0.30–0.22)	0.751	−0.04 (−0.09–0.08)	0.878	−0.19 (−1.92–1.53)	0.824
Model 3^d^	−0.04 (−0.34–0.25)	0.784	−0.02 (−0.12–0.07)	0.656	−0.37 −2.08–1.34)	0.670
Pattern 3						
Model 1^b^	−0.25 (–0.51–0.00)	0.05	-0.08 (–0.17–0.00)	0.051	1.13 (–0.61–2.88)	0.203
Model 2^c^	–0.23 (–0.49 –0.02)	0.080	–0.08 (–0.16–0.00)	0.069	0.81 (–0.90–2.53)	0.352
Model 3^d^	–0.22 (−0.52–0.06)	0.131	–0.08 (–0.18–0.01)	0.094	0.40 (–1.25–2.38)	0.835
Pattern 4						
Model 1^b^	–0.12 (–0.38–0.13)	0.348	–0.03 (–0.12–0.05)	0.411	0.61 (–1.14–2.37)	0.496
Model 2^c^	–0.09 (–0.35 –0.16)	0.477	–0.03 –0.11–0.05)	0.490	0.38 (–1.34–2.12)	0.660
Model 3^d^	–0.07 (–0.37 –0.22)	0.623	–0.01 (–0.12–0.08)	0.712	0.16 (–1.53–1.86)	0.849
Pattern 5						
Model 1^b^	–0.35 (–0.61 to –0.09)	0.008	–0.09 (−0.18 to –0.00)	0.033	1.82 (0.05–3.59)	0.043
Model 2^c^	−0.36 (−0.62 to −0.10)	0.007	−0.10 (−0.19 to –0.01)	0.022	1.92 (0.18–3.66)	0.030
Model 3^d^	–0.38 (–0.67 to–0.08)	0.012	–0.11 (–0.21 to –0.01)	0.027	1.78 (0.11 – 3.82)	0.047

^a^Beta regression coefficient; the positive B values indicated that higher adherence of nutrient patterns increase the higher changes in dependent variables and vice versa

^b^Adjusted for age and sex

^c^Adjusted for model 1 and body mass index, physical activity, and smoking (yes or no), energy intake, education levels (under diploma, diploma and associate degree, bachelor and higher), marital status (single, married), and employment status (employed, unemployed). For changes of insulin indexes, their values in baseline phase were adjusted

^d^Adjusted for models 1 and 2 and diabetes status at baseline and family history of diabetes

In age and sex adjusted model, per each quartile increment of the fifth nutrient pattern, the beta (β) coefficient (95 % CI) of changes in insulin, HOMA-IR, and HOMA-S were − 0.35 (− 0.61, − 0.09); P value = 0.008, -0.90 (-0.18, -0.00); P value = 0.033, and 1.82 (0.05, 3.59); P value = 0.043, respectively. In the fully adjusted model of the fifth pattern, the β coefficients (95 % CI) of changes in insulin, HOMA-IR, and HOMA-S were − 0.38 (− 0.67, − 0.08); P value = 0.012, -0.11 (-0.21, -0.01); P value = 0.027, and 1.78 (0.11, 3.82); P value = 0.047, respectively and remined significant. The present study showed no statistically significant relation between other nutrient patterns and the three-year changes in insulin related indices in different adjusted models.

## Discussion

In the present study, we extracted five nutrient patterns among participants of the population-based TLGS cohort study. Each quartile increment of the fifth pattern, characterized by fructose, vitamins A and C, pyridoxine, and potassium, associated with the reduced risk of hyperinsulinemia, IR, and insulin insensitivity. Other patterns did not show a significant relationship with the three years change in insulin-related markers.

There is no previous study investigating the association of nutrient patterns and insulin-related indices to our knowledge. Across quartiles of the first pattern, characterized by plant protein, thiamin, niacin, and minerals, carbohydrate and protein intake increased, and fat decreased. Fiber intake elevated due to the rise in grain and vegetable consumption, while fruit and dairy consumption decreased. Also, consistent with elevating this pattern’s score, other healthy foods, including white meat, legume, and nuts, have more consumed. Also, across this pattern, the intake of simple sugars and sweeteners was reduced due to increased nutrient density upward following the quartiles.

Plant protein was positively correlated with the first pattern. Although we expected the plant protein has a protective effect against IR, based on the literature [18], in our population, more supplied from refined grains (white bread and rice) which their role in the development of insulinemia and leading IR is demonstrated previously [19]. Also, thiamin and niacin are two appetite booster nutrients associated with more carbohydrate-rich foods [20]. This pattern also correlated with magnesium, zinc, and selenium. These minerals’ beneficial role was recognized; magnesium reduced diabetes and IR risk by stimulating insulin secretion by regulating ATP-sensitive potassium channels and the voltage-dependent calcium channels [21, 22]. Zinc under physiologic conditions is abundant in pancreatic islets and plays a role in insulin crystallization and secretion. Evidence also suggests that zinc regulates the glucose transporter GLUT4 translocation and glucose utilization by cells [23]. The protective effects of selenium are due to its antioxidant properties [24]. The number of diabetic patients, glucose-lowering drug consumption, and serum levels of glucose and insulin were increased across the quartiles of this pattern at the study’s baseline; so, before adjusting these variables, the overall effects of this pattern tended to elevate the risk of IR. However, after adjusting the diabetes status and family history of diabetes, the first pattern showed no significant relation with serum insulin, IR, and insulin sensitivity. A previous study showed that a driven nutrient pattern rich in thiamine, zinc, and plant protein is associated with significant reductions in glycated hemoglobin and fasting glucose [25]. This inconsistent result may be justified with differences in study design, derived nutrient pattern components, dietary habits, race, etc.

Higher adherence to the second nutrient pattern showed no association with changes in insulin-related indices. It seems that there is a neutral balance between beneficial and detrimental nutrients on IR in this pattern. The animal protein intake intensifies IR and reduced insulin sensitivity [26]. Calcium intake showed a protective association with IR and improved insulin sensitivity in studies [27]; however, in vitro studies showed that intracellular calcium above normal range disturbed insulin secretion and reduce insulin sensitivity [28]. On the other hand, dietary vitamin D and zinc were observed to reduce IR and reinforce insulin sensitivity. The association of other nutrients in this pattern with IR is unclear. This nutrient pattern is mostly correlated with dairy intake. Previous studies’ findings of the association between dairy consumption and IR are inconsistent [29–31]. Although a meta-analysis of clinical trials suggests that dairy intake, especially low-fat dairy, has a beneficial effect on HOMA-IR [30], high dairy intake is reported as a significant predictor of IR in some observational studies [29–31].

The third pattern tended to reduce the changes in insulin and HOMA-IR and was marginally significant. A higher score of this pattern had a positive correlation with vegetable intake. The vegetable consumption was 100 g/d higher in individuals in the highest compared to those in the lowest quartile; also, fiber, vitamin k, manganese, and potassium intake had high loading in this pattern. On the other hand, dietary iron intake had a high correlation with this pattern, which its effects triggering oxidative stress, lipid peroxidation, and IR has been documented [32]. Despite the expected beneficial roles of fiber [33], vitamin k [34], manganese [35], and potassium [36] on IR, which previously reported in studies, it seems that the combination with iron neutralize their impacts and attenuated the association of the third pattern and insulin and HOMA-IR changes.

The fourth nutrient pattern had a strong negative correlation with vitamin E, an antioxidant component, and beneficial dietary fats, including MUFA and PUFA; however, it was correlated with starch and folate. Previously reported, an extracted nutrient pattern, highly loaded in starch, is associated with higher weight gain [11]. Furthermore, two patterns, one with a highly loaded amount of carbohydrates, starch, and simple sugars [37] and another rich in fats, especially saturated fatty acids, increased the risk of obesity [38].

Vitamin E [39], MUFA [40], and folate intake [41] were related to improving insulin sensitivity and IR; however, starch [42] and in some studies, high RBC folate concentration [43] showed adverse effects on IR development. The most significant reduction in fat and increment in carbohydrate intake occurred across quartiles of this pattern; however, sweets and simple sugars were not reduced. Although vegetables, legumes, and nuts intake decreased due to increased fruit and grains intake, mostly refined grains, calorie intake remained stable across quartiles. Also, fat intake reduction was compensated by increasing the intake of simple carbohydrates. It seems that these interactions caused the association of this pattern with IR and insulin sensitivity not to be significant.

Higher adherence to the fifth pattern was negatively associated with insulin and HOMA-IR changes and showed a positive association with HOMA-S. This pattern was characterized by fructose, vitamins A and C, pyridoxine, and potassium. A recent study among Japanese adults showed a pattern rich in fiber, potassium, vitamins A, and C reduced metabolic syndrome risk [44]. The relationship of the fifth pattern components has been investigated with insulin and related indices previously. Briefly, vitamin A enhanced insulin sensitivity via upregulating the insulin receptors on the cell membrane [26]. Potassium supplementation reduced IR through decreasing interleukin 17-A (IL-17A) [45] and neutralized the adverse effects of high Na intake on IR development. In this pattern, intake of Na rich foods such as grains and red meat was significantly reduced, while fruit and vegetable consumption as major sources of potassium increased by 4 and 2 times, respectively, in the highest versus lowest quartile. The effect of the usual dietary vitamin C intake on IR is unknown, but it is shown that high serum levels of ascorbic acid and a 1000 mg/d vitamin C supplementation are associated with decreased IR and insulin levels and improvement in glycemic control [46]. Studies reported that pyridoxine has beneficial effects in diabetes control by reducing diabetes-related disruptions in dopaminergic receptors of liver islet cells and improving insulin signaling [47] and preventing endothelial dysfunction, IR, and intrahepatic fat accumulation [47]. Consistent with previous studies, high dietary fructose intake, as a low glycemic index sweetener, not merely has no adverse effects on insulin secretion stimulation but even it might be helpful for the management of type 2 diabetes [48]. However, some studies positively linked a high intake of fructose from sweetened beverages with IR [49].

Regarding the high correlation between fructose and Vitamin A, vitamin C, and potassium, it seems that fruits are the main source of fructose in our study. So, it is expected that the mentioned nutrients in the form of a dietary pattern have synergetic effects on each other and finally provide an efficient mixture to reduce IR and improve insulin sensitivity. Although studies showed a clear correlation between lower dietary fat intake and reduced risk of IR and diabetes, two important issues should be noted; firstly, decreasing fat intake in iso-caloric or limited energy diets does not necessarily improve diabetes. Secondly, how to replace fat with other dietary components and the type of carbohydrates and proteins consumed are determining factors.

Two previous meta-analyses showed that during the long-term intake of a low-fat diet (< 30 % energy), high protein intake had no significant effect on the glycemic control indices including fasting insulin, glucose, and glycosylated hemoglobin (HbA1c) compared with low or standard protein intake [50, 51]. This finding is consistent with our results, where the fat intake was reduced remarkably from quartiles 1 to 4 in pattern 1 (from 34.6 to 27.7) and pattern 4 (from 37.9 to 24.6), and consequently, protein significantly increased. However, we observed no beneficial association with insulin and IR, but we also showed that HOMA-IR was increased in people with higher adherence to the first pattern. The protein sources are another factor that was introduced as an effective agent for diabetes and IR risk. Several meta-analyses estimated that higher total and animal protein associated with a higher risk of diabetes [18, 52–55] and plant protein tended to reduce diabetes risk or observed no association [18, 52–55]. Studies mostly showed a direct association between red and processed meat and diabetes; however, there are inconsistent findings of other protein food sources such as dairy and egg. Meta-analyses showed that milk consumption reduces the risk of diabetes, though another fined it increase the risk [18, 54]. Inconsistent with previous studies, decreasing the red and processed meat intake across quartiles of pattern 5 in our study was related to lower fasting insulin and IR.

We observed that a balance is established between macronutrients (60.5 % carbohydrate, 30.5 % fat, 14 % protein) across quartiles of pattern 5. protein just increased by 0.5 % (from 13.4 to 13.9 %); this increment occurred in white meat, vegetables, nut, and legume sources and the red meat and dairy was decreased.

The present study has valuable strengths. It benefits from prospective design, relatively large sample size, and accurate data collection by trained interviewers versus previous studies that mostly used self-reported questionnaires. There are also some noticeable limitations. Although we adjust any possible confounders, some unknown confounders might affect the finding; also, since factor analysis was used to identify patterns, limitations of this method can be accounted for in our study. The number of derived factors is much dependent on the decisions of the researchers, which affect the factor loading of nutrients in each pattern [7]. However, we selected a wide range of main nutrients that may affect insulin homeostasis; additionally, five extracted patterns explained 62.2 % of the total variations in main nutrient intakes.

Insulin-related disorders are involved in developing a wide range of chronic diseases, and diet plays a major role in worsening or managing them. It makes sense to look at these disorders from a dietary aspect. This study suggests the potential role of diet for insulin-related disorders management from the nutrient patterns’ perspective. Furthermore, our study has clinical and public health applications. It means that if future studies confirmed the association observed in our study, it could be a scientific base for the production of dietary supplements containing a combination of nutrients proposed in the fifth pattern in this study.

## Conclusions

The present study’s findings showed that high adherence to a nutrient pattern characterized by vitamin A, vitamin C, pyridoxine, potassium, and fructose, mostly supplied from fruit and vegetables, is inversely associated with 3-years changes in insulin, HOMA-IR, and directly associated with HOMA-S. Our study was not shown any significant association between other patterns and changes in these insulin-related markers. These findings are consistent with the other studies’ results investigating similar disorders such as metabolic syndrome, diabetes, and obesity. As this study is the first one in this regard, it is necessary to be investigated in further study and other populations.

## Data Availability

The data analyzed in the present study are available by the corresponding author on reasonable request.

## Abbreviations

HOMA-IR: Homeostatic model assessment for insulin resistance
HOMA-S: Homeostatic model assessment for insulin sensitivity
HbA1c: Hemoglobin A1c
FBS: Fasting blood sugar
2-hBS: 2-hours blood sugar
IL-17A: Interleukin 17-A
SFA: Saturated fatty acids
MUFA: Monounsaturated fatty acids
PUFA: Polyunsaturated fatty acids
RBC: Red blood cells
ECLIA: Electrochemiluminescence immunoassay
USDA: United States department of agriculture
FCT: Food composition table
IR: Insulin resistance
BMI: Body mass index
FFQ: Food frequency Questionnaire
CI: Confidence interval
SD: Standard deviation
